# Effects of Rice Bran Oil Shortening Substitution on Physicochemical and Functional Properties of Plant-Based Mozzarella Cheeses

**DOI:** 10.3390/foods15081448

**Published:** 2026-04-21

**Authors:** Suteera Vatthanakul, Prapasri Theprugsa, Natchaya Jewsuwan, Witoon Prinyawiwatkul

**Affiliations:** 1 Department of Food Science and Technology, Faculty of Science and Technology, Thammasat University, Klong Luang, Pathumthani, Bangkok 12121, Thailand; prapasritheprugsa@gmail.com (P.T.); natchaya.jew@gmail.com (N.J.); 2 Center of Excellence in Food Science and Innovation, Thammasat University, Klong Luang, Pathumthani, Bangkok 12121, Thailand; 3 School of Nutrition and Food Sciences, Louisiana State University, Agricultural Center, Baton Rouge, LA 70803, USA; wprinya@lsu.edu

**Keywords:** mozzarella cheese, plant-based mozzarella cheese, rice bran oil, palm kernel oil, microstructure, physicochemical properties

## Abstract

Palm kernel oil is commonly incorporated into plant-based cheeses to mimic the textural and structural properties of animal fats owing to its high saturated fat content. Nevertheless, growing concerns regarding saturated fat consumption have stimulated research into alternative lipid sources for plant-based products. Therefore, this study aimed to evaluate the effects of substituting palm kernel oil with rice bran oil shortening (SRBO) on some selected physical, textural, functional, chemical, fatty acid and microstructural properties of plant-based mozzarella cheese analogs. Five formulations with SRBO levels of 0, 25, 50, 75, and 100% were prepared and their physicochemical properties were analyzed. Increasing SRBO significantly affected color due to natural pigments in rice bran oil. The pH value declined with higher SRBO, likely due to oxidation of unsaturated fatty acids. Texture profile analysis showed increases in hardness, springiness, cohesiveness, gumminess, and chewiness when SRBO was increased from 0% to 100%. Meltability slightly decreased at 25–75% but remained unchanged at 100% SRBO, while stretchability decreased significantly, attributed to β-type fat crystals disrupting protein networks. The work of shear decreased significantly (*p* ≤ 0.05), indicating improved spreadability attributed to the softer, less-crystalline nature of unsaturated fats compared to saturated fats. Proximate analysis revealed reduced fat content and a shift from saturated to unsaturated fats, notably oleic and linoleic acids, offering potential cardiovascular benefits. Confocal laser scanning microscopy showed denser fat crystal networks and smaller fat droplets at higher SRBO levels, enhancing oil retention and stability. Protein, fiber, moisture, and ash content remained stable across samples. These findings suggested that SRBO could be a functional and health-conscious alternative to palm kernel oil in plant-based mozzarella cheese, improving nutritional quality without compromising texture or functionality.

## 1. Introduction

Plant-based mozzarella cheese has gained popularity in the food industry due to the growing consumer trend toward plant-based diets. This shift in consumer behavior is influenced by various factors, including health-related dietary restrictions. Lactose malabsorption is widely prevalent across populations worldwide, affecting a substantial proportion of individuals [1], which may contribute to the increasing demand for lactose-free and plant-based dairy alternatives. Plant-based cheese, therefore, serves as a viable alternative for these individuals. As a result, the market for plant-based dairy alternatives, including cheese, has experienced continuous growth [2]. The plant-based cheese market is rapidly expanding, with a market size of USD 4.47 billion in 2025 and an expected growth to USD 8.62 billion by 2032, corresponding to a CAGR of 9.8% over 2025–2032. Mozzarella cheese accounts for the largest market share at 31.4% [3]. The availability of plant-based cheese has also diversified beyond traditional forms such as blocks, slices, and spreads, to include shredded products, whole blocks, wedges, and balls, reflecting a growing emphasis on convenience and product design variety [4]. However, the development of plant-based cheese products remains challenging, particularly in replicating the functional and sensory properties of traditional dairy cheese, such as meltability, stretchability, and texture, which are critical factors influencing consumer acceptance. Fat is among the key components affecting these properties as it plays a crucial role in determining texture and mouthfeel. In addition to meltability and texture, fat also significantly contributes to spreadability, a key quality attribute in mozzarella-style applications.

Palm kernel oil is commonly used in plant-based cheese formulations due to its ability to mimic the texture of milk fat. Nevertheless, palm oil is high in saturated fatty acids, which, when consumed in large quantities, may cause negative health impacts and increase the risk of non-communicable diseases (NCDs) [5]. Considering these concerns, reducing the intake of saturated fatty acids and replacing palm oil with other oils richer in unsaturated fatty acids, such as rice bran oil, presents a promising alternative. Rice bran oil not only contains healthier unsaturated fats but also provides bioactive compounds such as oryzanol, which is known for antioxidant properties, cholesterol-lowering effects and the potential to reduce the risk of cardiovascular diseases [6,7,8]. Studies have shown that rice bran oil shortening possesses desirable crystallization properties, which contribute positively to the textural characteristics of food products [9].

Plant-based cheese is a product designed to mimic the flavor and texture of dairy cheese while being entirely free of animal-derived ingredients. Its development has been ongoing to improve quality and achieve properties closer to those of dairy-based cheese. Plant-based cheese is typically an emulsion composed of plant-derived oils or fats and proteins, water, emulsifiers, colorants, flavorings, and other additives [10]. Several factors have driven increased consumer adoption of plant-based cheese, including lactose intolerance, cow milk allergy, a growing preference for vegetarian diets, and ethical concerns regarding animal welfare. Comparisons between dairy cheese and plant-based cheese reveal that plant-based alternatives generally contain lower energy and fat levels but provide less protein. Some plant-based cheeses may also have high sodium content, which could have negative implications for consumer health. Therefore, the nutritional quality of plant-based cheeses should be carefully considered prior to consumption [11]. Beyond flavor, texture, and nutritional composition, additional factors in plant-based cheese production include allergenicity (e.g., from nuts, peas, or other legumes), the use of preservatives, sodium content, and the incorporation of colorants [12]. Furthermore, challenges such as residual beany flavors and heat-stable anti-nutritional factors (ANFs), including lectins and trypsin inhibitors, may negatively impact both sensory attributes and nutritional quality [13].

Various strategies have been explored to improve the structural and functional properties of plant-based cheese. For example, the incorporation of hydrocolloids such as Sanxan gum has been shown to enhance texture, water- and oil-holding capacities, and microstructural uniformity, enabling plant-based cheese analogues to achieve properties comparable to dairy cheese [14]. In addition, fermentation using specific bacterial cultures has been applied to reduce off-flavors and modulate firmness in plant-based systems [15]. However, these approaches primarily focus on modifying protein or hydrocolloid interactions, while the role of lipid phase structuring remains relatively underexplored. This limitation is particularly important in mozzarella-type products, where meltability and stretchability are strongly governed by fat structure and its interaction with the surrounding matrix [16,17]. Conventional fats such as coconut and palm kernel oil provide desirable solid-like behavior but are associated with high saturated fat content. In contrast, liquid oils rich in unsaturated fatty acids (e.g., sunflower or canola oil) often lack the structural functionality required for cheese applications [17,18,19]. Structuring approaches such as oleogelation have been proposed; however, these systems typically rely on external structuring agents, which may complicate formulation and affect sensory quality.

To date, there is no existing research exploring how the specific polymorphic behavior and crystal organization of SRBO influence the microstructural development and heat-induced functional properties of plant-based mozzarella. Therefore, this study aimed to investigate SRBO as a structural substitute for palm kernel oil to develop plant-based mozzarella with functional properties similar to dairy cheese while providing a healthier lipid profile.

## 2. Materials and Methods

### 2.1. Materials

Raw materials and their sources included: cashew nuts (*Anacardium occidendium*; Siam Makro Public Co., Ltd., Bangkok, Thailand), soybeans (*Glycine max* (L.) Merrill; Raitip Co., Ltd., Bang Yai, Nonthaburi, Thailand), palm kernel oil (Patum Vegetable Oil Co., Ltd., Bangkok, Thailand), rice bran oil shortening (Thai Edible Oil Co., Ltd., Bangkok, Thailand), salt with 99.9% purity (Pure Salt Industry Co., Ltd., Bangkok, Thailand), glycerin and carrageenan (Krungthep Chemi Co., Ltd., Bangkok, Thailand), modified starch (SMS Corporation Co., Ltd., PathumThani, Thailand), non-dairy creamer color powder (R&B Food Supply Public Co., Ltd., Bangkok, Thailand), masking flavor and mozzarella cheese flavor (Givaudan (Thailand) Co., Ltd., Bangkok, Thailand) and egg yellow color (Tratazein & Sunset Yellow; FCF) food coloring (Best odour Co., Ltd., Bangkok, Thailand).

### 2.2. Plant-Based Milk Preparation

Cashew nut milk was prepared following a previously reported method with slight modifications [20]. First, cashew nuts were separately cleaned and boiled for 20 min. The boiled cashew nuts were weighed and blended (Philips, Model HR2221/00, Bangkok, Thailand) with clean water at a ratio of 1:4 (*w*/*v*). Then, the mixture was strained to remove residues, and the extracted liquid was boiled at 100 °C for 20 min. The same procedure was applied for the preparation of soybean milk.

### 2.3. Plant-Based Mozzarella Cheese Preparation

Plant-based mozzarella cheese was prepared using a blend of cashew nut milk and soybean milk with texture-modifying agents and other ingredients through dissolving, mixing, and heating, as follows: First, salt (1.5%), carrageenan (0.4%), and water (31.65%) were added to mixture of soybean milk and cashew nut milk at a ratio of 1:1.5 (26%), as shown in Table 1. The mixture was stirred until fully dissolved and homogeneous.

Subsequently, the solution was transferred to a blender (Philips, Model HR2221/00, Bangkok, Thailand), followed by the addition of modified starch (20.8%) and non-dairy creamer color powder (0.4%). The mixture was blended at speed level 2 for 1 min.

Next, the fat phase was added to obtain a total fat content of 18% (*w*/*w*), consisting of palm kernel oil and rice bran oil shortening (SRBO) at different substitution levels (0%, 25%, 50%, 75%, and 100%, *w*/*w*), along with glycerin (1%). The total fat content was kept constant across all formulations, with palm kernel oil progressively replaced by SRBO on a weight basis. This design allowed the effect of fat type and crystallization behavior to be evaluated independently of total fat content.

Blending was continued for 1 min to obtain a homogeneous mixture. The mixture was then transferred to a heating vessel, where color and flavoring agents were added and mixed until evenly dispersed. The mixture was heated to 80 °C for 5 min with continuous stirring. After heating, the product was poured into PPE plastic containers and stored at −18 °C for 8–12 h to allow proper setting. The samples were subsequently stored at 4 °C until further analysis.

Five formulations were prepared by varying the substitution level of palm kernel oil with SRBO, as shown in Table 2, while all other ingredients were kept constant.

### 2.4. Physicochemical Measurement

#### 2.4.1. pH

The pH of the cheese samples was measured by weighing 10 g of cheese and homogenizing it in 100 mL of distilled water for 1 min. The pH value was determined using a pH meter (Boqu, Model PH-26-6, Shanghai, China).

#### 2.4.2. Color

Color measurement was conducted on cheese samples that were cut into 20 mm cubes (width × length × thickness). The color was evaluated using the CIE Lab system, where L* indicated lightness, a* indicated redness, and b* indicated yellowness. A Hunter Lab colorimeter (Hunter, Model CX2687, Bridgeton, MO, USA) was used for the measurements.

#### 2.4.3. Water Activity

Water activity was measured by cutting 5 g of cheese into small pieces and placing them into a container. The a_w_ value was then determined using a water activity meter (AquaLab, Model CX2, Pullman, WA, USA).

#### 2.4.4. Texture Profile Analysis

The Texture Profile Analysis (TPA) was performed to analyze the texture structure. Samples were prepared using a cylindrical die cutter with a 20 mm diameter, then trimmed to 20 mm in height. All samples were kept at 5 °C and analyzed within 5 min after cutting. The sample disks were analyzed using a TA.XTPlus texture analyzer (Stable Micro Systems, Model TA.XTPlus, Godalming, UK) equipped with a 75 mm cylindrical plate and 30 kg load cell. The samples were compressed to 50% of their original height at a crosshead speed of 1.00 mm/s with a 5 s interval between compressions. The data were recorded in grams and analyzed using Exponent software version 8.

#### 2.4.5. Stretchability, Meltability and Spreadability

Stretchability analysis was performed using a Texture Analyzer. A 100 g cheese sample was heated in a microwave oven (Panasonic, Model NN-ST342N, Bangkok, Thailand) at 660 watts for 3 min. Subsequently, the samples were tested for texture properties using a fork probe. The instrument was configured with a 10 kg load cell, a test speed of 10 mm/s, a post-test speed of 10 mm/s, and a distance of 270 mm.

For meltability analysis, cheese was filled into a test tube with a diameter of 16 mm. The initial height of the cheese was measured using a vernier caliper before refrigerating the sample at 4 °C for 4 h. The test tube was then placed horizontally in a water bath (Memmert, Model WNB14, Schwabach, Germany) at 100 °C for 100 min. After heating, the test tube was cooled in an ice water bath (0–2 °C) for 5 min. The length of the melted cheese was measured in cm. The melting distance was determined by calculating the difference between the initial height of the cheese before heating and the length of the melted cheese after heating.

Spreadability analysis was performed following a methodology adapted from a previous study [21]. using a TA.XTPlus texture analyzer equipped with a spreadability rig (HDP/SR) and a 5/25 kg load cell (Stable Micro Systems). Shear work was quantified as the total force exerted during the spreading process, wherein lower values corresponded to reduced force requirements and thereby indicated enhanced spreadability. Samples were carefully placed within a female cone possessing a 90° angle. The compression distance was standardized at 23 mm, with a testing speed of 3 mm/s and a post-test speed of 10 mm/s. All samples were evaluated immediately upon removal from refrigeration at 4 °C to ensure consistent temperature conditions.

#### 2.4.6. Proximate Composition and Fatty Acid Composition Analyses

Proximate analysis including crude protein, fat, dietary fiber, moisture and ash was determined according to the AOAC method [22]. The chemical composition of the plant-based mozzarella cheese product was reported on a % wet basis.

Fatty acid composition of the cheese samples was analyzed using gas chromatography (GC) with an Agilent 7890B instrument at the Institute of Food Research and Product Development (IFRPD), Kasetsart University. Approximately 50 g of cheese was prepared and placed in clean, airtight containers that protected the samples from light and moisture. The samples were stored at a low temperature (4 ± 2 °C) during transportation to prevent lipid oxidation. Fatty acid analysis was performed using the GC system, and the results were reported as the content of individual fatty acids in g per 100 g of cheese.

#### 2.4.7. Confocal Laser Scanning Microscopy (CLSM)

The microstructure of plant-based mozzarella cheese was analyzed using confocal laser scanning microscopy (CLSM) (Zeiss, Model LSM800, Oberkochen, Germany) at a magnification of 10×. A Nile Red staining solution was prepared at a concentration of 0.1 g/L in ethanol. A small amount of cheese was placed onto a microscope slide using a stainless-steel spatula and evenly spread into a thin film. Then, 30 µL of the Nile Red solution was applied to the sample and evenly distributed before being covered with a cover slip. The prepared slide was stored in an icebox at 4 ± 2 °C for 5–10 min prior to imaging. Nile Red was excited using a laser wavelength of 488 nm, and the stained lipid regions appeared red under CLSM.

### 2.5. Statistical Analysis

Data were expressed as mean ± standard deviation (SD). All formulations were prepared in three independent batches (biological replicates), and each analysis was performed in triplicate (technical replicates). The experiment was designed as a completely randomized design (CRD). Statistical differences among treatments were evaluated using one-way analysis of variance (ANOVA). Prior to analysis, the assumptions of normality and homogeneity of variance were assessed using the Shapiro–Wilk test and Levene’s test, respectively. When the assumption of homogeneity of variance was violated (*p* ≤ 0.05), Welch’s ANOVA was applied as an alternative method. When significant differences were detected (*p* ≤ 0.05), means were separated using Duncan’s multiple range test for data meeting the assumption of homogeneity of variance, while Games–Howell post hoc test was used for data with unequal variances. All statistical analyses were performed using IBM SPSS Statistics version 28.0.

## 3. Results

### 3.1. pH, Water Activity and Color

The varying ratios of palm kernel oil to rice bran oil shortening significantly (*p* ≤ 0.05) influenced the pH values of the plant-based mozzarella cheese. Overall, an increase in SRBO substitution led to a general decline in pH values, ranging from 6.02 to 5.25 (Table 3). This decline may be attributed to the high content of unsaturated fatty acids in SRBO, which are susceptible to oxidation when exposed to heat during processing [23]. Such oxidative reactions produce free fatty acids (FFAs), which are acidic in nature and consequently lower the pH of the product as the amount of unsaturated fats increases [24]. The water activity (a_w_) values ranged from 0.94 to 0.99. Regarding the color parameters, the substitution of palm kernel oil with SRBO resulted in appearance of sample. Although some fluctuations were observed, the incorporation of SRBO generally resulted in lower L* values compared with control (0% SRBO), while redness (a*) and yellowness (b*) significantly increased. This was primarily attributed to the inherent pigment profiles of the lipid sources. Rice bran oil contains natural pigments, such as carotenoids, which absorb light in the blue-green spectrum and reflect longer wavelengths, leading to deeper color intensity and higher a* and b* values [25]. In contrast, palm kernel oil often undergoes a bleaching process during refining, which removes these pigments and results in a lighter appearance. Consequently, the reduction in palm kernel oil in the formulation led to a decrease in the overall lightness of the final product.

### 3.2. Texture

Texture profile analysis (Table 4) of mozzarella cheese analogs formulated with different substitution levels of rice bran oil shortening revealed statistically significant variations in pre-heating textural properties (*p* ≤ 0.05). Increasing the SRBO from 0% to 75% in the plant-based mozzarella cheese formulation caused a reduction in texture hardness from 1123 g to 685 g (Table 4). This was primarily because SRBO is a liquid oil rich in unsaturated fatty acids, which lack the rigid crystalline structure found in solid saturated fats like palm oil. Unlike saturated fats that form a solid fat crystal network, SRBO remains liquid which may have acted more like a lubricant, increasing spreadability while decreasing texture hardness, leading to a softer, more compliant structure. In contrast, when the SRBO increased to 100%, an increase in hardness was observed. This trend can be explained by differences in fat crystallization behavior; rice bran oil shortening tends to promote the formation of β (beta) crystals, characterized by a larger size, dense packing, and high thermal stability. Such crystal morphology contributes to a firmer, more stable, and more compact product structure [26] at low temperatures compared to a palm oil blend. In contrast, palm kernel oil typically crystallizes in the β′ (beta-prime) form, which consists of finer crystals that yield a softer texture. This has been confirmed using X-ray diffraction (XRD) and differential scanning calorimetry (DSC) [27]. The increasing hardness observed with increasing rice bran oil shortening aligns with previous research indicating that the hardness of rice bran oil-based oleogels increases with higher beeswax content [28]. These results reinforced the notion that both fat composition and crystal morphology play critical roles in determining textural firmness.

Springiness also exhibited an increasing trend with higher SRBO content (Table 4). This enhancement may reflect the well-dispersed crystalline network within the fat gel matrix, which facilitates the formation of a resilient three-dimensional structure. A uniform crystal distribution is known to improve elasticity and shape recovery after deformation, thereby enhancing springiness [29]. Conversely, cohesiveness significantly decreased (*p* ≤ 0.05) in the formulation with 100% SRBO. This reduction was likely due to the high proportion of unsaturated fatty acids in plant-based fats, which weakens the interactions between fat and protein molecules. As a result, the internal structure becomes more fragile and less cohesive [30]. Interestingly, chewiness and gumminess reached their maximum values in the sample formulated with 100% SRBO (Table 4). This outcome suggested that the increases in hardness and springiness exert a dominant influence on these parameters. Despite the reduction in molecular-level binding forces, the presence of a robust and elastic network yields a gel-like texture with pronounced chewiness and adhesive mouthfeel [31]. Both parameters increased significantly in the fully substituted formulation (100% SRBO).

The variability in TPA values observed at different concentrations of %SRBO can be attributed to multiple factors. Random sampling for TPA may have encompassed regions of the cheese with differing levels of structural uniformity. Moreover, the mixing method significantly influenced the microstructure. In this study, a blender was employed, which provided relatively low shear force. This limited shear capacity may have hindered uniform dispersion of the fat phases, leading to slower crystallization, the formation of larger crystals, and heterogeneous distribution within the cheese matrix, thereby contributing to fluctuations in TPA measurements. In contrast, homogenizers deliver higher shear forces that promote finer dispersion of fat droplets and the formation of fat crystals with more uniform sizes [32]. Similar findings have been reported in previous studies [33], which demonstrated that non-homogenized cheeses lacking emulsifying salts developed large fat clusters and heterogeneous textures, whereas homogenized samples exhibited more consistent crystal structures. Collectively, these results highlight the importance of homogenization in achieving uniform fat crystallization and reducing variability in texture measurements, whereas blending increases the likelihood of structural heterogeneity and inconsistent TPA outcomes.

### 3.3. Meltability and Stretchability

The functional properties of plant-based mozzarella cheeses formulated with varying levels of SRBO after heating (Table 5) were largely influenced by the characteristics of the continuous fat particles embedded in the cheese matrix, which dictate melting behavior and textural changes upon heating [34,35]. In plant-based cheese systems, fat droplets are typically dispersed within a continuous matrix of plant proteins, starch, hydrocolloids and water; the complex interactions among these constituents ultimately determine the final functional attributes [35,36,37].

The results showed that meltability tended to significantly decrease up to 75% SRBO, followed by an increase at 100% SRBO. In contrast, stretchability as represented by Resistance to Extension showed a decreasing trend with increasing proportions of SRBO. Similar observations have been reported in previous studies indicating that plant-based cheeses exhibited markedly lower meltability and stretchability compared to dairy-based cheeses (*p* < 0.05) [37]. This difference is mainly attributed to the absence of casein micelle structures that normally form the elastic protein network responsible for melting and stretching behavior in dairy cheeses [17,38,39]. In addition, the protein concentration in plant-based cheese formulations is often lower than that of dairy cheese, which may limit the ability of the protein phase to form a continuous and elastic network capable of supporting melt and stretch properties. Although fats with lower melting points are theoretically expected to melt more readily during heating and enhance mobility within the protein matrix, thereby improving meltability and stretchability through plasticizing effects and enhanced interactions between protein and fat [39], an opposite trend was observed in this study, which has also been reported in previous studies [40]. This phenomenon may be explained by the crystallization behavior of fats present in SRBO. Such fats tend to form β type crystals characterized by dense and stable structures [41], which can disrupt protein bonding within the continuous phase and hinder the formation of a cohesive and elastic network [42,43]. Consequently, intermolecular interactions within the matrix cannot organize into a stable structure [44,45], leading to reduced stretchability and elasticity. These findings support previous reports indicating that the structural characteristics of fat particles may exert a greater influence on meltability than the melting point of the fat itself [46]. Therefore, despite SRBO having a lower melting point than palm kernel oil, the reduced meltability and stretchability observed in this study are likely associated with its crystalline fat structure. Another explanation may involve the structural role of starch and hydrocolloids within the continuous phase. Plant based cheese frequently rely on starch-based matrices to compensate for the absence of casein networks. During heating and cooling, starch undergoes gelatinization followed by partial retrogradation, forming a three-dimensional polysaccharide network capable of entrapping water and fat droplets [16,44]. As the SRBO level increased, the resulting matrix may have become denser and more rigid, thereby restricting fat mobility and limiting the lubricating effect of the fat phase despite the relatively high unsaturated fatty acid content of the oil [47].

The work of shear showed a statistically significant decrease (*p* ≤ 0.05) as presented in Table 5, indicating improved spreadability of the product. This behavior is likely related to the higher proportion of unsaturated fatty acids present in rice bran oil shortening. Unsaturated fats generally form fewer solid crystals at low temperatures, resulting in softer and smoother textures compared with saturated fats, which tend to form dense crystalline structures that reduce spreadability [48]. Consequently, the incorporation of SRBO likely contributed to a softer matrix that required less energy for deformation, resulting in improved spreadability.

The reduced stretchability observed with increasing SRBO is likely influenced by multiple interacting factors. Fat crystallization behavior appears to play a dominant role, as the formation of β-type crystals may disrupt matrix continuity and limit elastic deformation. In addition, the relatively low protein content in the formulation may not be sufficient to form a strong, continuous network comparable to that in dairy cheese, which is essential for stretchability. Instead, the continuous phase is primarily governed by starch and hydrocolloids, which form a viscoelastic gel matrix. Upon heating, starch retrogradation and hydrocolloid interactions may increase structural rigidity and reduce molecular mobility, thereby further limiting melt flow and stretchability. These combined effects suggest that both fat structure and matrix composition contribute to the observed behavior, with fat crystallization acting as the primary controlling factor.

### 3.4. Proximate Composition

According to the proximate composition analysis (Table 6), the substitution of palm kernel oil with SRBO resulted in a slight reduction in fat content from 20.51% to 18.25%. This slight reduction may be attributed to minor lipid losses or variations during the manufacturing process. Meanwhile, moisture, crude protein, crude fiber, and ash contents did not differ significantly (*p* > 0.05) among all formulations. The protein content of the plant-based mozzarella samples remained consistently low, ranging from 0.74% to 0.78%, which is considerably lower than that of traditional dairy cheese (approximately 23.0%). Such nutritional differences between plant-based and dairy cheese are commonly reported [49]. Dairy cheese typically contains higher levels of protein and calcium (approximately 20.7% protein and 568.1 mg calcium per 100 g), as well as essential vitamins such as B6, B9, and B12, which play important roles in neurological and cognitive functions. In contrast, the nutritional composition of plant-based cheese depends largely on the raw materials used, with most formulations providing less than 5% protein [50]. Even plant-based products with relatively higher protein levels, such as vegan tofu, contain only about 13.3% protein and 17.8 mg calcium per 100 g. Therefore, although plant-based cheeses generally provide lower nutritional value than dairy cheese, they remain a viable alternative for individuals with lactose intolerance or cow milk allergy.

### 3.5. Fatty Acid Composition

The fatty acid composition of plant-based mozzarella cheese samples, with varying levels of SRBO replacing palm kernel oil, is presented in Table S1. Increasing the substitution level from 25% to 100% SRBO resulted in a progressive reduction in saturated fatty acids (SFA), specifically lauric acid (C12:0), myristic acid (C14:0), and palmitic acid (C16:0). Conversely, the proportions of unsaturated fatty acids (UFAs), including oleic acid (C18:1), linoleic acid (C18:2), and alpha-linolenic acid (C18:3), increased accordingly. These results demonstrate a clear shift in the lipid profile from a saturated-dominant system to one enriched with unsaturated fats, thereby potentially reducing the dietary intake of saturated fats. In addition to the improved fatty acid profile, the incorporation of SRBO introduces essential bioactive compounds, including γ-oryzanol, tocotrienols and phytosterols. These constituents exhibit potent antioxidant activities and play a vital role in modulating lipid metabolism [51]. Dietary intake of rice bran oil has been associated with improved serum lipid profiles through the reduction in total cholesterol, low-density lipoprotein cholesterol (LDL-C), and triglycerides [52]. Furthermore, γ-oryzanol and phytosterols can effectively inhibit intestinal cholesterol absorption and enhance bile acid excretion, further contributing to cardiovascular health and systemic lipid homeostasis [53]. Furthermore, the replacement of palm kernel oil with rice bran oil shortening leads to a reduction in saturated fatty acids and an increase in unsaturated fatty acids, which is consistent with current dietary recommendations aimed at reducing the risk of non-communicable diseases. These findings suggest that the developed plant-based mozzarella cheese not only provides desirable functional properties but also has the potential to offer improved nutritional value.

### 3.6. Microstructure of Plant-Based Mozzarella Cheese

**Figure 1:**
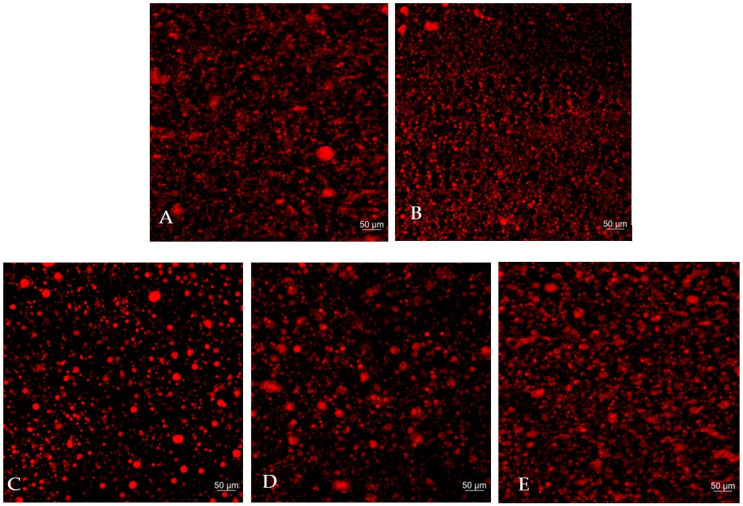
Confocal laser scanning microscopy (CLSM) image of plant-based mozzarella cheese at different substation level: (**A**) 0% SRBO, (**B**) 25%SRBO, (**C**) 50%SRBO, (**D**) 75%SRBO and (**E**) 100%SRBO. 10× magnification of all samples. Red fluorescence represents the lipid phase.

The microstructure of plant-based mozzarella cheese formulated with varying substitution levels of palm kernel oil by SRBO was analyzed using Confocal Laser Scanning Microscopy (CLSM) at 10× magnification, as shown in Figure 1. The red coloration indicates the distribution of fat droplets within the samples. Analysis revealed that plant-based mozzarella cheese with 0% and 100% SRBO (Figure 1A,E) exhibited uniformly distributed fat droplets with similar morphology. This suggests that the use of a single fat source results in a more homogeneous microstructure compared to samples containing mixed fat types (the β′ (beta-prime) crystals of palm kernel oil and the β (beta) crystals of rice bran oil shortening). These findings are consistent with previous studies indicating that plant-based cheese samples formulated with 100% coconut oil displayed a more uniform microstructure than those formulated with blends of coconut and sunflower oils, attributed to differences in fat crystal structures [54].

Although samples with 25%, 50%, and 75% SRBO levels (Figure 1B–D) showed more heterogeneous fat droplet morphology, they still demonstrated greater fat-rich region connectivity than samples without rice bran oil shortening. This indicates that the amount of rice bran oil shortening plays a crucial role in forming a denser fat crystal network. This observation aligns with previous studies reporting that increasing fat concentration led to the formation of longer, more fibrous crystals and a more extensive crystalline network [19,55]. Therefore, increasing the proportion of SRBO promoted the development of stronger fat crystal networks, which may enhance oil retention within the cheese matrix. Additionally, small spherical fat globules were observed, encapsulated by the continuous phase appearing as dark regions composed of water, starch, and hydrocolloids [41]. This continuous phase mimics the role of casein proteins in dairy cheese by preventing fat droplet coalescence and phase separation, thereby maintaining uniform fat dispersion throughout the product [42,56]. The fat globules are dispersed as spherical entities within the cheese structure, which is predominantly composed of fat and water. Hence, plant-based cheese could be characterized as an oil-in-water (O/W) emulsion, where fat is dispersed in a matrix of water, starch, and proteins, contributing to product stability and texture [15,55,57].

## 4. Conclusions

This study demonstrates that rice bran oil shortening (SRBO) can be used as an alternative to palm kernel oil in plant-based mozzarella cheese, resulting in an improved fatty acid profile with higher levels of unsaturated fats. Beyond compositional changes, the findings highlight that fat crystallization behavior and microstructural organization play a critical role in determining product functionality. In particular, the formation of β-type fat crystal networks contributed to increased firmness while limiting meltability and stretchability, providing new insights into structure–function relationships in plant-based cheese systems. However, this study has several limitations. The absence of sensory evaluation limits the assessment of consumer acceptance, and storage stability as well as long-term quality changes were not investigated. Future research should include sensory and consumer studies to validate product acceptance, as well as shelf-life evaluation covering microbial stability, lipid oxidation, and color and textural changes during storage. Overall, these findings provide a scientific basis for optimizing fat design in plant-based cheese and contribute to the development of healthier and more functional dairy alternatives.

## Figures and Tables

**Table 1:** Formulations of plant-based mozzarella cheese.

Ingredients	Percentage
Cashew nut milk	15.6
Soybean milk	10.4
Salt	1.5
Fat	18.0
Carrageenan	0.4
Modifieds starch	20.8
Non-dairy creamer color powder	0.4
Glycerin	1.0
Water	31.65
Flavor	0.14
Color	0.11

**Table 2:** The ratio of palm kernel oil and rice bran oil shortening in different formulations.

Sample Codes	Percentage	
	**Palm Kernel Oil**	**Rice Bran Shortening (SRBO)**
0% SRBO	100	0
25% SRBO	75	25
50% SRBO	50	50
75% SRBO	25	75
100% SRBO	0	100

**Table 3:** pH, water activity and color values of plant-based mozzarella cheese.

Samples *	pH	Water Activity (a_w_)	L*	a*	b*
0% SRBO	6.02 ^a^ ± 0.01	0.99 ^a^ ± 0.01	81.84 ^a^ ± 0.01	−0.50 ^e^ ± 0.01	25.29 ^e^ ± 0.10
25% SRBO	5.42 ^c^ ± 0.08	0.98 ^a^ ± 0.01	80.42 ^c^ ± 0.01	0.70 ^a^ ± 0.01	30.54 ^a^ ± 0.47
50% SRBO	5.67 ^b^ ± 0.00	0.95 ^b^ ± 0.00	80.22 ^d^ ± 0.00	0.12 ^d^ ± 0.01	26.47 ^d^ ± 0.03
75% SRBO	5.68 ^b^ ± 0.23	0.97 ^a^ ± 0.01	81.27 ^b^ ± 0.01	0.24 ^c^ ± 0.02	26.94 ^c^ ± 0.04
100% SRBO	5.25 ^c^ ± 0.02	0.94 ^b^ ± 0.02	79.29 ^e^ ± 0.02	0.27 ^b^ ± 0.00	27.84 ^b^ ± 0.60

Values were expressed as mean ± standard deviation (*n* = 3). Means in the column with different superscripts letters (a–e) are significantly different (*p* ≤ 0.05) according to Duncan’s multiple range test. The samples represent the percentage of rice bran oil shortening (SRBO) used to replace palm kernel oil. * See Table 1 for sample description.

**Table 4:** Texture properties of plant-based mozzarella cheese.

Samples *	Hardness (g)	Springiness (mm)	Cohesiveness	Chewiness (g)	Gumminess (g)
0% SRBO	1223.07 ^a^ ± 128.01	0.57 ^d^ ± 0.02	0.46 ^d^ ± 0.14	290.48 ^d^ ± 2.49	545.29 ^c^ ± 14.45
25% SRBO	1204.20 ^a^ ± 273.96	0.71 ^b^ ± 0.04	0.73 ^ab^ ± 0.44	469.40 ^b^ ± 11.82	663.11 ^b^ ± 15.51
50% SRBO	675.41 ^b^ ± 105.45	0.73 ^b^ ± 0.01	0.74 ^a^ ± 0.01	297.92 ^c^ ± 1.54	409.68 ^d^ ± 5.83
75% SRBO	685.60 ^b^ ± 118.18	0.65 ^c^ ± 0.23	0.66 ^b^ ± 0.01	229.83 ^e^ ± 5.66	362.53 ^e^ ± 30.79
100% SRBO	1290.66 ^a^ ± 402.31	0.77 ^a^ ± 0.01	0.54 ^c^ ± 0.02	549.76 ^a^ ± 8.10	689.52 ^a^ ± 10.20

Values were expressed as mean ± standard deviation (*n* = 3). Means in the column with different superscripts letters (a–e) are significantly different (*p* ≤ 0.05) according to Games–Howell post hoc test. The samples represent the percentage of rice bran oil shortening (SRBO) used to replace palm kernel oil. * See Table 2 for sample description.

**Table 5:** Meltability, resistance to extension, stretch quality and work of shear of plant-based mozzarella cheese.

Samples *	Meltability (cm)	Resistance to Extension (g_f_)	Stretch Quality (g/mm)	Work of Shear (g/mm)
0% SRBO	3.35 ^b^ ± 0.49	2557.82 ^a^ ± 2.09	2.60 ^a^ ± 0.05	426.79 ^a^ ± 5.68
25% SRBO	4.25 ^a^ ± 0.50	2546.66 ^a^ ± 40.79	0.74 ^b^ ± 0.02	415.08 ^b^ ± 4.22
50% SRBO	3.35 ^b^ ± 0.77	2233.56 ^b^ ± 29.07	0.35 ^c^ ± 0.01	389.22 ^c^ ± 5.86
75% SRBO	3.90 ^ab^ ± 0.28	1877.20 ^c^ ± 29.30	0.81 ^b^ ± 0.01	375.48 ^d^ ± 4.88
100% SRBO	3.90 ^ab^ ± 0.51	1871.66 ^c^ ± 19.33	0.28 ^c^ ± 0.53	347.78 ^e^ ± 6.82

Values were expressed as mean ± standard deviation (*n* = 3). Means in the column with different superscripts letters (a–e) are significantly different (*p* ≤ 0.05) according to Duncan’s multiple range test. The samples represent the percentage of rice bran oil shortening (SRBO) used to replace palm kernel oil. * See Table 2 for sample description.

**Table 6:** Proximate composition of plant-based mozzarella cheese.

Samples *	Moisture	Fat	Protein	Fiber	Ash
0% SRBO	43.31 ^a^ ± 0.29	20.51 ^a^ ± 0.28	0.75 ^a^ ± 0.03	3.07 ^a^ ± 0.84	2.50 ^a^ ± 0.92
25% SRBO	42.73 ^b^ ± 0.07	19.79 ^b^ ± 0.26	0.74 ^a^ ± 0.00	3.12 ^a^ ± 0.16	2.73 ^a^ ± 0.78
50% SRBO	42.81 ^b^ ± 0.01	19.53 ^b^ ± 0.07	0.74 ^a^ ± 0.01	3.06 ^a^ ± 0.89	2.66 ^a^ ± 0.45
75% SRBO	42.65 ^b^ ± 0.94	18.76 ^c^ ± 0.24	0.78 ^a^ ± 0.02	3.18 ^a^ ± 0.21	2.44 ^a^ ± 0.14
100% SRBO	43.99 ^a^ ± 0.02	18.25 ^c^ ± 0.37	0.76 ^a^ ± 0.00	3.10 ^a^ ± 0.07	2.72 ^a^ ± 0.01

Values were expressed as mean ± standard deviation (*n* = 3). Means in the column with different superscripts letters (a–c) are significantly different (*p* ≤ 0.05) according to Duncan’s multiple range test. The samples represent the percentage of rice bran oil shortening (SRBO) used to replace palm kernel oil. * See Table 2 for sample description.

## Data Availability

The original contributions presented in this study are included in the article/supplementary material. Further inquiries can be directed to the corresponding author.

## Supplementary Materials

The following supporting information can be downloaded at: https://www.mdpi.com/article/10.3390/foods15081448/s1, Table S1: Fatty acid composition of plant-based mozzarella cheeses.
