# Supplementary material for: Effects of Rice Bran Oil Shortening Substitution on Physicochemical and Functional Properties of Plant-Based Mozzarella Cheeses
Source: Foods. 2026 Apr 21;15(8):1448. doi: 10.3390/foods15081448 (PMC13114895; doi:10.3390/foods15081448)
Supplement: Supplementary file 1 [file foods-15-01448-s001.zip › foods-4197643-supplementary.pdf]

Table S1. Fatty acid composition of plant-based mozzarella cheeses.

Fatty acids (g/100 g)	Samples*				
	**0% SRBO**	**25% SRBO**	**50% SRBO**	**75% SRBO**	**100% SRBO**
Butyric acid C4:0	Not detected	Not detected	Not detected	Not detected	Not detected
Caproic acid C6:0	0.05	0.04	0.03	0.02	Not detected
Caprylic acid C8:0	0.65	0.50	0.36	0.22	0.06
Capric acid C10:0	0.57	0.45	0.32	0.19	0.06
Lauric acid C12:0	7.49	5.79	3.88	2.08	0.09
Myristic acid C14:0	2.67	2.09	1.43	0.80	0.12
Palmitic acid C16:0	4.84	4.01	3.10	2.34	1.63
Stearic acid C18:0	0.46	0.52	0.58	0.65	0.71
Tran-Elaidic acid C18:1trans	0.02	0.02	0.01	0.03	0.03
Oleic acid C18:1, Omega 9	3.32	3.98	4.69	5.65	6.38
Linoleic acid (C18:2, Omega-6)	0.59	1.54	2.55	3.74	4.80
Alpha-Linolenic acid (C18:3, ALA)	0.01	0.05	0.08	0.13	0.17
Arachidi acid (C20:0)	0.03	0.07	0.12	0.17	0.22
Eicosenic acid (C20:1)	0.02	0.04	0.06	0.08	0.09
Butyric acid C4:0	Not detected	Not detected	Not detected	Not detected	Not detected

* The samples represent the percentage of rice bran oil shortening (SRBO) used to replace palm kernel oil.
